# Expression of Aldehyde Dehydrogenase (ALDH1) and ATP Binding Cassette Transporter G2 (ABCG2) in Iraqi Patients with Colon Cancer and the Relation with Clinicopathological Features

**DOI:** 10.22088/IJMCM.BUMS.7.4.234

**Published:** 2019-03-30

**Authors:** Noah Abdulqader Mahmood, Zaynab Saad Abdulghany, Israa Mahdi Al-Sudani

**Affiliations:** 1 *Molecular Biology Department, Iraqi Center for Cancer and Medical Genetics Research, Mustansiriyah University, Baghdad, Iraq. *; 2 *Medical College, Ibn Sina University, Baghdad, Iraq. *

**Keywords:** Colon cancer, Iraqi patients, ALDH1, ABCG2, immunohistochemistry

## Abstract

Tumor initiation cells or cancer stem cell markers ABCG2 and ALDH1 play pivotal roles in invasion, metastasis and resistance to cytotoxic agents. In this study, we evaluated the expression levels of ALDH1 and ABCG2 in Iraqi patients with colon cancer and/or benign colon tumors. We also investigated the association between the expression levels of these markers and patient clincopathological features. The expression levels of ALDH1 and ABCG2 in cancer tissues as well as in benign tumor samples were determined by immunohistochemistry using tumor tissues microarray of TNM (Tumor, Node, Metastasis) in 42 patients with colon cancer samples as well as in 18 corresponding benign tumors. Immunohistochemistry showed that ALDH1 and ABCG2 expression levels increased to 80% and 76%, respectively in colon cancer tissues as compared to 33% and 28% in benign tumor tissues. The expression levels of ALDH1 and ABCG2 were associated with tumor stages. No significant association was found between the expressions levels of these markers and tumor size, gender, patients' age, and lymph node involvement. These results indicate that the expression levels of ALDH1 and ABCG2 increased in colon cancer tissues compared to benign tumor tissues in Iraqi patients.

Recently, growing proof has suggested that human cancers may result from a small number of cells called tumor initiation cells or cancer stem cells (CSCs) (1, 2). These cells had the capacity to form and sustain new tumor in immune-deficient mice since they have the ability to self-renewal and differentiation. The expression of potential biomarkers of CSCs was studied extensively to understand their mechanism of action in cancer grow, progress and response to conventional therapy (3). These cells may be generated from disturbances in the renewal process of stem cells (4), or may result either from somatic mutations in normal tissue stem cells that convert those normal cells into malignant cells or from additional differentiation of progenitor or overripe cells that gain the capability to self-renewal in addition to tumorigenic abilities (5). Conventional chemo-radiotherapy agents may reduce the volume of tumor without affecting CSCs. Therefore, patients may suffer from cancer retrogression. These cells can be identified and separated from different solid tumors by using putative CSC markers (6). In this study, the expression levels of CSC markers aldehyde dehydrogenase 1 (ALDH1) and ATP-binding cassette super-family G member 2 (ABCG2) were determined in Iraqi patients with colon cancer, and the association between the expression levels of these markers and tumor stages, lymph node involvement, patient's age and gender were examined.

## Materials and Methods

**Patients and tumor characteristics**

This study involved 42 colon cancers as well as 18 benign tumors surgically harvested from patients admitted to Alyarmook Teaching Hospital in Baghdad, Iraq between November 2016 and September 2017. Ethics committee approval was received for this study from Iraqi Center for Cancer and Medical Genetics Research/ Mustansiriyah University law No. 160/91. All participants have signed informed consents before donating their tissue samples and undergoing surgery, into the legal of the above mentioned hospital. All patients with colon cancer recruited in this study were having no chemotherapy or radiation therapy. The mean age of patients was 57 years (range was 22- 80 years). The colonic patients diagnosed with colon cancer were recruited and diagnosed according to American Joint Committed on Cancer (AJCC) and/or according to duke's classification (Table 1). While patients diagnosed with other diseases with colon problems (non-cancerous) were recruited as benign colon tissues. Total colostomy, hemicolectomy and/or colonic mass biopsy were used in the diagnosis of patient's tumors. Ten of the colon cancers were diagnosed as stage I (24%) while 19 cases were diagnosed as stage II colon cancer (45%). The other 13 colon cancers were diagnosed as stage III colon cancer (31%). Fresh colon cancer tissues, as well as benign tissue samples were collected and fixed in 10% formalin and embedded in paraffin to be used in immunohistochemistry staining.

**Table 1:** Clinicopathological features of patients with colon cancer

**Parameters**	**Patients (n)**	**Percentage (%)**
Age of patients		
≤50 years ≥50 years	17 25	40 60
Gender		
Women Men	23 19	55 45
Tum or size		
T1 T2 T3 T4	8 20 10 4	19 48 24 9
Tumor stage		
Stage I Stage II Stage III	10 19 13	24 45 31
Lymph node metastasis		
Positive Negative	10 32	24 76

**Immunohistochemistry staining**

Immunohistochemistry staining was carried out using standard staining technique. Four macrometer of paraffin embedded sections were cut and mounted on positive charge glass slides and de-paraffinized in xylene and rehydrated in serial alcohol solution. The sections were treated with heat induced retrieval antigen (HIRA) in citrate buffer at 121 °C for 15 min, and washed twice. After endogenous peroxidase blocking with 3% hydrogen peroxide, slides were incubated with. bovine serum albumin (BSA) and then incubated with anti-ALDH1 antibody (A1334-33W US-bio, USA) and/or anti-ABCG2 antibody (Sc-3`77176 Santa Cruz Biotechnology, USA) overnight at 4 °C. Secondary antibody (DAKO Biotechnology, USA) was added followed by incubation with avidin-biotin for 30 min at room temperature. Slides were treated with diaminobenzidine (DAB), countered with hematoxylin, dehydrated and mounted with water-free mounting medium (DPX), than analyzed by light microscope

**Staining evaluation**

The number of stained cells and the staining intensity of both ALDH1 and ABCG2 were taken together. Normal cells were scored as zero (no positive staining), score 1 (when 1-10% of the cells were positive staining), score 2 (when 11-50% of the cells were positive staining) and score 3 (when 51-100 % of the cells were positive staining). The positive intensity was considered as zero (none), weak positive, moderate positive and strong positive. Both zero and score 1 were considered as low expression and both scores 2 and 3 were recorded as high expression.

**Statistical analysis**

To evaluate the relationship between protein expression levels of ALDH1 and ABCG2 and patients, clinicopathological features, chi-square (χ^2^) statistic has been used to investigate whether expression values differ between colon cancer tissues and benign tumors. P<0.05 was considered as statistically significant.

## Results

**Protein expression of ALDH1 and ABCG2**

**Fig. 1:**
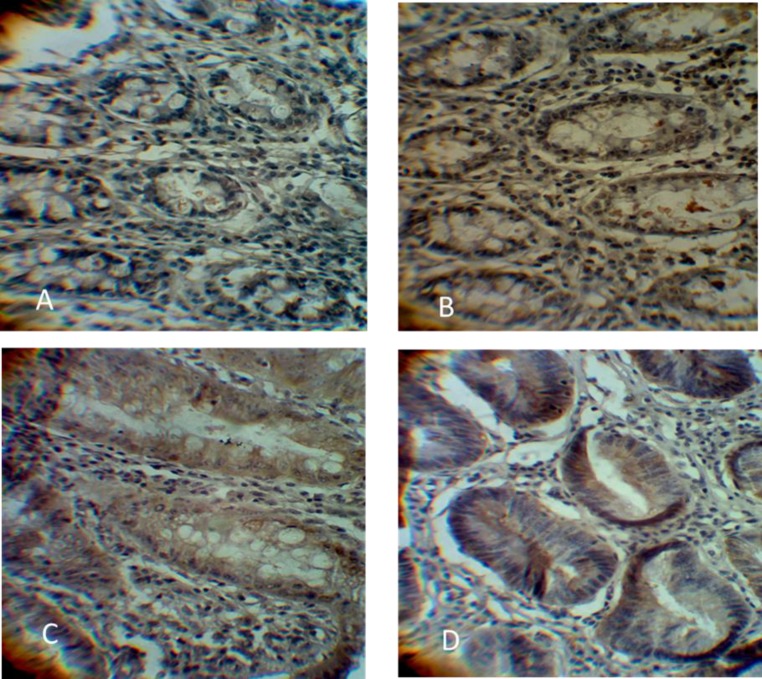
Immunohistochemistry staining of ALDH1 protein in both benign and cancereous colon tissues. A: low ALDH1 protein expression in benign tissues; B: stage I colon cancer showing positive ALDH1 in the cytoplasm of the cells; C: stage II colon cancer with increased ALDH1 expression level; D: stage III colon cancer with high ALDH1 expression. All images were magnified at 400X

**Fig. 2.**
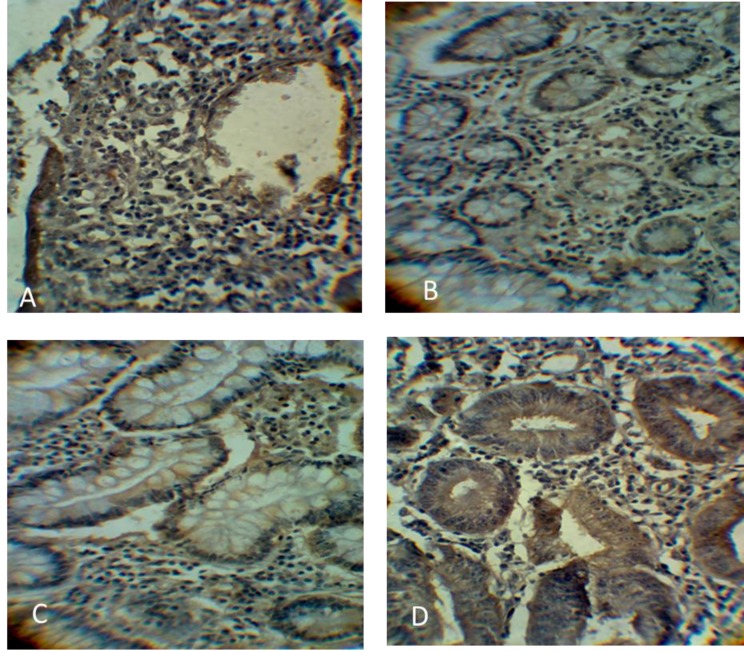
**Immunohistochemistry staining of ABCG2 protein in both benign and cancererous colon tissues.** A: low ABCG2 protein expression in benign tissues; B: stage I colon cancer showing positive ABCG2 in cytoplasm and cell membrane; C: stage II colon cancer with increased ABCG2 expression level; D: stage III colon cancer with high ABCG2 expression in tissues. All images were magnified at 400X

Immunohistochemistry data showed that ALDH1 and ABCG2 levels were higher in colon cancer in comparison with benign tumors (Table 2). ALDH1 was mainly expressed in the cytoplasm, while ABCG2 was expressed in the cytoplasm and cell membrane of the cells. Immunostaining revealed that ABCG2 and ALDH1 markers were highly expressed in 76.2% and 80.0% of cancerous tissues as compared to 28% and 33% of benign tumor tissues, respectively. The expression of ALDH1 and ABCG2 proteins in colon tumor stages are shown in figures 1 and 2, respectively.

**Relationship between ALDH1 and ABCG2 expression levels and patient clinical features**

The results of biological markers are presented in table 3, ALDH1 expression level was significantly associated with tumor stages (P=0.01). There was no significant association between ALDH1 expression level and tumor size, lymph node,

gender, and/or patient's age. On the other hand, the results showed a positive association between ABCG2 expression level and tumors stages (P= 0.025) and negative association between ABCG2 expression level and tumor size, lymph node, gender and /or patients' age.

**Table 2:** ALDH1 and ABCG2 expression levels in colon cancer tissues (n=42) and benign tumors (n=18)

**Expression**		**High expression** **n (%)**	**Low expression** **n (%)**	**P value**
**ABCG2**	**Benign tissues**	5 (28)	13 (72)	0.03 *
	**Cancer tissues**	32 (76)	10 (24)	
**ALDH1**	**Benign tissues**	6 (33)	12 (67)	0.001 *
	**Cancer tissues**	34 (80)	8 (20)	

* Statistically significant (P< 0.05)

**Table 3:** Association between ALDH1 and ABCG2 expression and clinicopathological parameters

**Pathological** **data**	**Patients number**	**ALDH1**			**ABCG2**		
		**High expression**	**Low expression**	**P-value**	**High expression**	**Low expression**	**P-value**
**Age of patient**							
**≤50 years** **>50 years**	17 25	11 (65%) 17 (68%)	6 (35%) 8 (32%)	0.82	9 (53%) 14 (56%)	8 (47%) 11 (44%)	0.84
**Gender**							
**Women** **Men**	23 19	14 (61%) 12 (63%)	9 (39%) 7 (37%)	0.87	10 (43%) 6 26%)	13 (57%) 13 (74%)	0.42
**Tumor size**							
**T1** **T2** **T3** **T4**	8 20 10 4	3 (38%) 12 (60%) 8 (80%) 4 (100%)	5 (62%) 8 (40%) 2 (20%) _Not detected_	0.11	2 (25%) 11 (55%) 7 (70%) 4 (75%)	6 (75%) 9 (45%) 3 (30%) 1 (25%)	0.16
**Tumor stage**							
**Stage I** **Stage II** **Stage III**	10 19 13	3 (30%) 13 (68%) 11(85%)	7(70%) 6(32%) 2(15%)	0.01 *	4 (40%) 14 (74%) 11 (84%)	6 (60%) 5 (28%) 2 (26%)	0.025*
**Lymph node metastasis**							
**Positive** **Negative**	10 32	9 (90%) 21 (66%)	1 (10%) 11 (34%)	0.13	8 (80%) 17 53%)	2 (20%) 15 (47%)	0.13

* Statistically significant (P <0.05)

## Discussion

Iraqi population registered low rate of colon cancer incidence but with a steady increase according to population increase over time (7). The connotation of CSCs has been suggested since two decades (4). The most important characteristics of these cells are tumorigenicity enhancement and the ability for self-renewal and/or differentiation (8). Thus, these cells may lead to the formation of new tumors with high efficiency, but also containing both CSC and non- CSC populations. Moreover, most CSCs exhibit resistance to conventional anti-cancer therapies such as chemotherapy and radiotherapy (9). To date, CSCs have been identified in various malignancies, including acute myeloid leukemia (10), brain tumors (11), hepatocellular carcinoma (12), breast, lung, pancreatic, and ovarian cancers (13-16). One of the most frequent methods to identify CSCs was instituted as dependence on the activity of membrane ATP-binding cassette (ABC) drug transporters, which mediate the efflux of Hoechst 33342 from the cytoplasm (17). Study of CSCs in solid tumors showed that cells with low levels of Hoechst 33342 dye (referred to as the subpopulation (SP) when separated by florescent activated sorting cells), might be highly tumorigenic (18). There is evidence indicating that due to the enhanced drug efflux, which is also mediated by ABC transporters, tumor SP cells are more resistant to the chemotherapeutic drugs than non-SP cells.

On the other hand, it is well known that many cancers are not sensitive to conventional chemotherapy, and this is in particular correct in colon cancer. Previous studies showed that high ALDH1A1 expression lead to chemotherapy resistance in cancer cell lines (19, 20). The results of these studies suggested that ALDH1A1 plays a pivotal role in chemotherapy resistance and could provide important information to understand the mechanism of conventional chemotherapy in solid tumors. Moreover, study on CSCs showed that the cells that expressed high level of ALDH1A1 can produce new tumors and metastasis when injected into NOD/SCID mice (21).

In this study, we found that ALDH1 was highly expressed in colon cancer tissues as compared to benign samples, and this may result from the differences in genetic or environmental factors such as toxic substances that affect ALDH1 expression. A previous study showed that the elevated expression level of ALDH1A1 was correlated with poor prognosis in colon cancer patients (22).

Several studies mentioned the expression level of membranous ABCG2 in colorectal tumors, and suggested that it could be used to predict post-operative patient's survival as prognostic biomarker (23). While Nielsen et al. concluded that the biological role of ABCG2 in clinical drug resistance is still unknown but ABCG2 protein expression is related to prognosis and drug prediction, suggesting the necessity of an international effort to standardize ABCG2 measurement (24).

In conclusion, the present study showed that ALDH1 was highly expressed in colon cancer tissues in comparison with benign tumor samples. In our study, high expression level of ALDH1 was linked with tumor stage, and this may explain the aggressiveness of colon cancer. On the other hand, results showed negative association between the ALDH1 expression level and other patient clinicopathological features. The non-significant association between ALDH1 expression level in the present study and other clinicopathological features could be attributed to the small sample size. Moreover, results showed that protein expression of ABCG2 was elevated in colon cancer tissues compared with non-cancerous tissues. Together, these results suggest that ALDH1 and ABCG2 may be used as biochemical markers in diagnosis and prognosis of colon cancer.
